# Inequalities in Smoking and Quitting-Related Outcomes Among Adults With and Without Children in the Household 2013–2019: A Population Survey in England

**DOI:** 10.1093/ntr/ntab211

**Published:** 2021-10-11

**Authors:** Loren Kock, Jamie Brown, Lion Shahab, Harry Tattan-Birch, Graham Moore, Sharon Cox

**Affiliations:** 1 Department of Behavioural Science and Health, University College London, London, UK; 2 SPECTRUM Research Consortium, Edinburgh, UK; 3 DECIPHer, School of Social Sciences, Cardiff University, Cardiff, UK

## Abstract

**Introduction:**

Smoking among those who live with children is an important influence on smoking initiation among children. This study assessed socioeconomic inequalities in smoking and quitting-related outcomes among all adults with and without children in the household.

**Aims and Methods:**

Monthly repeat cross-sectional household survey of adults (16+) from 2013–2019 in England (*N* = 138 583). We assessed the association between cigarette smoking and quitting-related outcomes and having children in the household, and whether these relationships were moderated by occupational social grade (categories AB–E from most to least advantaged). Trends in smoking prevalence among adults with and without children in the household were explored.

**Results:**

In adjusted analysis, the association of having children in the household with smoking prevalence depended on social grade: smoking prevalence was between 0.71 (95% confidence interval 0.66–0.77) and 0.93 (0.88–0.98) times lower among social grades AB–D with children in the household relative to those without. Conversely, it was 1.11 (1.05–1.16) times higher among social grade E. Yearly prevalence declined similarly among those with and without children (both prevalence ratio: 0.98, 95% confidence interval 0.97–0.99). Motivation to stop smoking was higher among those with children than those without, but lower among disadvantaged than more advantaged groups. Social grades D–E had greater heavy smoking, but higher prevalence of past-month quit attempts.

**Conclusions:**

Among the most disadvantaged social grade in England, smoking prevalence was higher in those with children in the household than without. To attenuate future smoking-related inequalities, there is an urgent need to target support and address barriers to quitting and promote longer-term quit success.

**Implications:**

In the most disadvantaged occupational social grade, having children in the household was associated with higher smoking prevalence compared with not having children. This contrasts with all other social grades in which there was lower comparative smoking prevalence among those with than without children in the household. Without attention this disparity could exacerbate existing and future health inequalities related to smoking.

## Introduction

The UK government has stated an ambition for England to be “smoke free” by 2030, defined as an overall tobacco smoking prevalence of less than 5%.^1^ To achieve this, there must be an acceleration of the progress seen on both smoking initiation and smoking cessation.^2^

Familial smoking and exposure to environmental tobacco smoke are associated with initiation and future smoking behavior among children.^3,4^ Progress in reducing current smoking prevalence among 11–15 year olds in England (22% in 1996 to 5% in 2018^5^) is likely partially reflective of the declines in adult smoking seen over a period of major tobacco control legislation (namely the 2007 smoke-free law, 2012 and 2015 tobacco retail display bans, 2015 ban on smoking in cars with children, 2016 standardized packaging and ongoing tobacco taxation).^6–9^ Despite this overall reduction, in recent years there is some evidence that the decline in smoking among youth is stalling across the UK nations,^5,10,11^ and a socioeconomic gradient in adult smoking remains.^12^ Those of more disadvantaged socioeconomic position (SEP) exhibit persistently higher smoking rates (25% in routine and manual occupations smoke vs. 10% in managerial and professional occupations) and bear a disproportionate burden of the associated morbidity and mortality.^13^

Following Hovell’s behavioral ecological model,^14^ a confluence of broad environmental determinants of health, including greater exposure to tobacco smoke and availability of cigarettes,^3,15^ family smoking role models,^16^ cultural smoking norms,^17^ and difficult or stressful life circumstances,^18^ children from disadvantaged households are more likely to become smokers compared with more advantaged children.^19^ Considering that most smokers in the United Kingdom start smoking before the age of 20,^20^ reducing the proportion of children and young people from disadvantaged households exposed to smoking by adults would likely reduce inequalities in smoking among young people and therefore reduce future adult or parental smoking.^21^ However, the outcomes of policies on parental smoking reduction can be patterned by SEP, with children from more disadvantaged families experiencing relatively smaller declines in exposure to smoking.^6,22^ This suggests that a broader set of social determinants likely undermine quit success.

The presence of children in the household may itself influence parental or adult quitting. While previous research has suggested that having children in the house did not consistently predict making a quit attempt or success,^23^ since the publication of this work it is possible that more recent changes in smoking norms in front of children and the further decline in smoking rates may motivate more parents or adults living with children to quit. However, recent smoking and quitting-related behavior and changes in smoking over time have not been examined in detail according to whether adults live with children, and whether socioeconomic circumstances moderate this relationship.

By determining the smoking environment in homes with children, adult smoking, and quitting-related outcomes remains an important target to reduce smoking-related inequalities. Using a nationally representative repeat cross-sectional survey in England, this study aimed to assess inequalities in smoking and quitting-related outcomes (current smoking, heaviness of smoking, motivation to stop smoking, and past-month quit attempts) among all adults in recent years by children in the household. We also aimed to explore trends in smoking prevalence between 2013 and 2019 according to SEP among adults with and without children in the household, respectively.

## Methods

### Sample and Recruitment

Data were drawn from the ongoing Smoking Toolkit Study (STS), a monthly repeated cross-sectional survey of a representative sample of adults (aged 16+) in England. Each month, a form of random location in combination with quota sampling is used to select a new sample of approximately 1700 adults aged 16 years and older. Further details on the design of the STS, including sampling technique can be found elsewhere.^24^ Sample weighting uses the rim (marginal) weighting technique to match the English sociodemographic population profile relevant to the time each monthly survey was collected. Thus, respondents with characteristics that are under-represented receive a larger weight, while those who are over-represented receive a smaller weight. Comparisons with sales data and other national surveys show that the STS recruits a representative sample of the population in England with regard to key demographic variables and smoking indicators.^24^

The sample dataset consisted of STS respondents from March 2013 to December 2019 inclusive. This timeframe was selected because STS respondents were first asked questions about having children in the household from March 2013 onwards.

### Measures

Specific wording for each measure is provided in Supplementary Materials.

### Dependent Variables

#### Current Smoking

Smoking status was ascertained using the following question and response options:

“Which of the following applies to you?”

1. I smoke cigarettes (including hand rolled) every day
2. I smoke cigarettes (including hand rolled), but not every day
3. I do not smoke cigarettes at all, but I do smoke tobacco of some kind (eg, Pipe, Cigar, or Shisha)
4. I have stopped smoking completely in the last year
5. I stopped smoking completely more than a year ago
6. I have never been a smoker (ie, smoked for a year or more)

Respondents were classified as current cigarette smokers (answers of 1 or 2 above) or former/nonsmokers (answers of 4, 5, and 6). Those who indicated that they do not smoke cigarettes but do smoke tobacco of some kind (answer 3) were excluded (0.4%) from the analysis because they do not complete equivalent measures of dependence to cigarette smokers (cigarettes per day and time to first cigarette after waking).

#### Smoking and Quitting-Related Outcomes

##### Heaviness of Smoking

Heaviness of smoking was measured using the heaviness of smoking index (HSI).^25^ The HSI uses two questions from the Fagerström Test for Cigarette Dependence: time to first cigarette in the morning after waking and the number of cigarettes smoked per day. An HSI score >4 is considered heavy smokers, and those with <4 considered to be lighter/moderate smokers.

##### Motivation to Stop Smoking

Motivation to stop smoking was assessed using the Motivation To Stop Scale,^26^ a single-item measure with seven response options representing increasing motivation to quit:

1. “I don’t want to stop smoking”
2. “I think I should stop smoking but don’t really want to”
3. “I want to stop smoking but haven’t thought about when”
4. “I REALLY want to stop smoking but I don’t know when I will”
5. “I want to stop smoking and hope to soon”
6. “I REALLY want to stop smoking and intend to in the next 3 months”
7. “I REALLY want to stop smoking and intend to in the next month”

For ease of interpretation, responses were collapsed into two variables reflecting high^7,8^ versus low^2–6^ motivation to stop smoking.

Consistent motivation to stop smoking has been shown to be a useful signal of recent smoking cessation attempts^27^ and was measured using responses to the question “Have you consistently felt that you wanted to stop smoking in the past year?” with answer responses of “No” or “Yes.”

##### Quit Attempts

Quit attempts in the past month were measured among current smokers using the question “How many serious attempts to stop smoking have you made in the last 12 months?” and if one or more attempts were reported: “How long ago did your most recent serious quit attempt start?” We distinguished those who attempted to quit up to 1 month ago versus those who made no quit attempt or attempted to quit more than 1 month before the survey interview but were not successful.

### Independent Variables

#### Children in the Household

Whether or not respondents have children at home was derived from a question regarding household status.

#### Social Grade

Occupation-based social grade was operationalized as the measure of SEP.^28^ The measure comprises AB (higher and intermediate managerial, administrative and professional), C1 (supervisory, clerical and junior managerial, administrative and professional), C2 (skilled manual workers), D (semi-skilled and unskilled manual workers), and E (state pensioners, casual and lowest-grade workers, unemployed with state benefits).

#### Time (Year)

Time was included as a continuous independent variable and comprises seven data points (the years 2013 [March onwards], 2014, 2015, 2016, 2017, 2018, 2019).

#### Sociodemographic Covariates

The sociodemographic characteristics sex (women or other), age (16–24, 25–34, 35–44, 45–54, 55–64, and ≥65 years), and region in England (London, North East, North West, Yorkshire and Humber, East Midlands, West Midlands, East of England, South East, and South West) were also included.

### Sample Selection

Overall, 139 323 (unweighted) adults aged 16+ were surveyed. Those who exclusively smoked cigars and pipes (*n* = 650) and responded with “Don’t know” to the question on smoking status (*n* = 90) were excluded. This left a final unweighted sample size for analysis of 138 583 adults.

### Analysis

Weighted descriptive statistics (% [*n*]) were used to report the variables included in the analyses.

#### Research Question 1

To assess the overall association between current cigarette smoking and having children in the household, we conducted a multivariable log-binomial regression model (unweighted), adjusted for age, sex, region, and year.

#### Research Question 2

Power analyses using simulations with STS data indicated that we were unlikely to have sufficient statistical power to assess realistic three-way interactions between having children in the household (Yes vs. No [referent]) and SEP (five categories of social grade: AB [referent], C1, C2, D, and E) and year (continuous) (see Supplementary Figure S4).

Therefore, a second unweighted model was run that included a two-way interaction term between having children in the household (Yes vs. No [referent]) and SEP (five categories of social grade: AB [referent], C1, C2, D, and E).

#### Research Question 3

To assess the associations between current smoking prevalence and year among adults with children in the household, we constructed a multivariable log-binomial regression model (unweighted) including year (continuous from 2013 to 2019) and SEP (five categories with social grade AB as referent) and the interaction term as explanatory variables. The inclusion of the SEP by year interaction term allowed us to examine any differential time trends according to SEP. We repeated this analysis among a sample of adults without children in the household.

The results from all models are reported as prevalence ratios (PRs) with 95% confidence intervals (CIs) adjusted for age, sex, region, and year. Participants with missing data for any of the variables in the analyses were excluded (*n* = 38 responses from smokers for the question on motivation to stop smoking). The Strengthening the Reporting of Observational Studies in Epidemiology (STROBE) reporting guideline was used in the design and reporting of this study.

The analysis plan and statistical code for this study were uploaded and made publically available on the open science framework (OSF) https://osf.io/6zt7x/ on 03/01/2021 before analyses were run. However, following feedback on the correct preregistration procedure during peer review, the study was formally registered on OSF 05/07/2021 (osf.io/xrdz7).

### Sensitivity Analyses

Housing tenure is a strong socioeconomic predictor of smoking in England.^29^ The same models were conducted but with housing tenure as an alternative measure of SEP^30^ and comprised the collapsed groups “Social housing” (local authority/housing association) and “Other” (mortgage bought, owned outright, private renting, and other).

A sensitivity analysis was also conducted using consistent motivation to stop as an alternative measure to the motivation to stop smoking scale.

## Results

A weighted total of 138 633 adults (mean [SE] age = 47.1 [0.05]; 51% women) completed the STS survey between 2013 and 2019. Among the overall sample, 17.8% were current cigarette smokers and 30.7% reported having children in the household. See Table 1 for an overview of sample characteristics.

**Table 1.** Characteristics of Total Sample (Weighted Data)

Characteristic	*N* = 138 633^a^
Current cigarette smoker	24 638 (17.8%)
Children in household	42 572 (30.7%)
Social grade	
AB	37 594 (27.1%)
C1	38 459 (27.7%)
C2	29 702 (21.4%)
D	20 731 (15.0%)
E	12 147 (8.8%)
Age	
16–24	19 644 (14.2%)
25–34	23 282 (16.8%)
35–44	22 637 (16.3%)
45–54	23 966 (17.3%)
55–64	19 539 (14.1%)
65+	29 565 (21.3%)
Gender	
Men/other	67 752 (48.9%)
Women	70 881 (51.1%)

^a^Unweighted *n* = 138 583.

### Cigarette Smoking

In the model without interactions (Table 3, Model 1), those with children in the household were less likely to smoke compared with those without (PR: 0.92, 95% CI 0.89–0.94; *p* < .001). Among all respondents, there was a social gradient in smoking prevalence, with higher rates among more disadvantaged social grades (Supplementary Table S1 and Table 2, Model 1).

**Table 2.** Association Between Cigarette Smoking Prevalence and Whether or Not There Are Children in the Household and Social Grade

Variable	Model 1 (without interaction)			Model 2 (interaction)		
	(χ ^2^(4) = 4560.7, *p* < .001)^a^			(χ ^2^(4) = 109.4, *p* < .001)^b^		
	PR^c^	95% CI^d^	*p*	PR^c^	95% CI^d^	p
Children in household						
No	—	—		—	—	
Yes	0.92	0.89, 0.94	**<.001**	0.71	0.66, 0.77	**<.001**
Social grade						
AB	—	—		—	—	
C1	1.59	1.53, 1.66	**<.001**	1.51	1.44, 1.59	**<.001**
C2	2.23	2.14, 2.32	**<.001**	2.06	1.97, 2.17	**<.001**
D	2.67	2.56, 2.78	**<.001**	2.46	2.34, 2.59	**<.001**
E	3.57	3.42, 3.72	**<.001**	3.10	2.95, 3.27	**<.001**
Children in household × social grade						
Yes **×** C1				1.19	1.09, 1.31	**<.001**
Yes **×** C2				1.29	1.18, 1.42	**<.001**
Yes **×** D				1.31	1.19, 1.44	**<.001**
Yes **×** E				1.56	1.42, 1.71	**<.001**

Values in bold are significant at p< .05.

Models adjusted for age, sex, region, and year.

^a^Result of likelihood ratio test comparing model with social grade variable includes without social grade variable.

^b^Result of likelihood ratio test comparing model with children in household and social grade interaction term with model without interaction term.

^c^Prevalence ratio.

^d^Confidence interval.

**Table 3.** Associations Between Smoking and Quitting-Related Outcomes and Whether or Not There Were Children in the Household and Social Grade

Variable	Motivation to stop smoking (without interaction)			Motivation to stop smoking (interaction)			Heaviness of smoking (without interaction)			Heaviness of smoking (interaction)			Past-month quit attempt (without interaction)			Past-month quit attempt (interaction)		
	χ ^2^(4) = 67.7, *p* < .001^a^			χ ^2^(4) = 1.78, *p* = .78^b^			χ ^2^(4) = 388.5, *p *< .001^a^			χ ^2^(4) = 5.8, *p *= .21^b^			χ ^2^(4) = 292.7, *p *< .001^a^			χ ^2^(4) = 4.64, *p* = .32^b^		
	PR^c^	95% CI^d^	p	PR^c^	95% CI^d^	p	PR^c^	95% CI^d^	p	PR^c^	95% CI^d^	p	PR^c^	95% CI^d^	p	PR^c^	95% CI^d^	p
Children in household																		
No	—	—		—	—		—	—		—	—		—	—				
Yes	1.18	1.10, 1.26	**<.001**	1.12	0.95, 1.32	.17	0.98	0.91, 1.05	.57	0.75	0.57, 0.99	**.047**	1.03	0.93, 1.14	.58	0.79	0.59, 1.04	.095
Social grade																		
AB	—	—		—	—		—	—		—	—		—	—		—	—	
C1	0.93	0.85, 1.02	.11	0.92	0.82, 1.03	.14	1.20	1.05, 1.38	**.008**	1.12	0.96, 1.31	.15	1.45	1.25, 1.69	**<.001**	1.32	1.10, 1.59	**.003**
C2	0.80	0.72, 0.88	**<.001**	0.76	0.67, 0.86	**<.001**	1.70	1.50, 1.94	**<.001**	1.55	1.33, 1.80	**<.001**	1.87	1.60, 2.19	**<.001**	1.68	1.39, 2.05	**<.001**
D	0.70	0.63, 0.78	**<.001**	0.69	0.60, 0.79	**<.001**	1.99	1.75, 2.27	**<.001**	1.87	1.61, 2.18	**<.001**	2.42	2.06, 2.85	**<.001**	2.21	1.81, 2.70	**<.001**
E	0.75	0.67, 0.83	**<.001**	0.74	0.65, 0.85	**<.001**	2.53	2.23, 2.89	**<.001**	2.39	2.07, 2.77	**<.001**	3.58	3.04, 4.21	**<.001**	3.17	2.60, 3.87	**<.001**
Children × social grade																		
Yes **×** C1				1.03	0.85, 1.25	.76				1.32	0.96, 1.82	.092				1.33	0.96, 1.85	.092
Yes **×** C2				1.13	0.92, 1.39	.24				1.43	1.06, 1.96	**.022**				1.36	0.97, 1.91	.075
Yes **×** D				1.06	0.86, 1.32	.57				1.29	0.95, 1.76	.11				1.32	0.94, 1.86	.11
Yes × E				1.04	0.83, 1.29	.75				1.27	0.94, 1.73	.13				1.43	1.01, 2.02	**.043**

Values in bold are significant at p< .05.

Models adjusted for age, sex, region, and year.

^a^Result of likelihood ratio test comparing model with social grade variable includes without social grade variable.

^b^Result of likelihood ratio test comparing model with children in household and social grade interaction term with model without interaction term.

^c^Prevalence ratio.

^d^Confidence interval.

**Figure 1.**
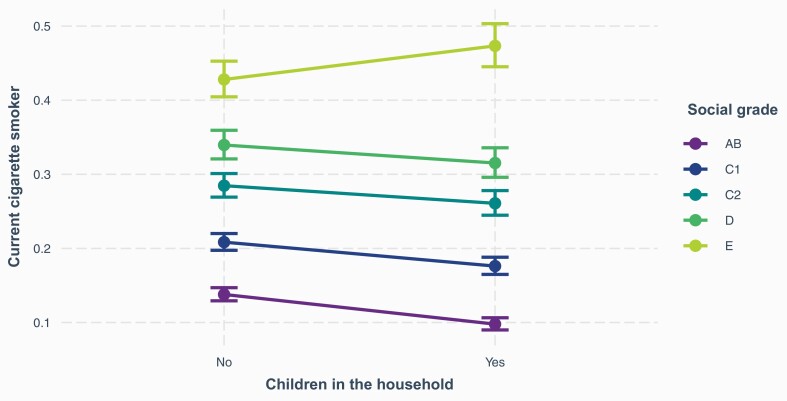
Interaction plot for smoking prevalence among adults with children in the household by social grade.

In the model including the interaction between children in the household and SEP (Table 2, Model 2), the association between cigarette smoking and social grade depended on whether or not respondents had children in the household. Figure 1 shows that differences in smoking prevalence between social grades are apparent when there are no children in the household. However, the disparities are even greater among those with children in the household. The moderation was greatest in the most disadvantaged social grade E. As a result, smoking prevalence was 0.71 times lower in AB participants with children in the household relative to those in AB without children (and 0.84, 0.92, and 0.93 times lower in C1, C2, and D participants, respectively). Conversely, it was 1.11 times higher in E participants with children in the household relative to those without children (Supplementary Table S2).

In a sensitivity analysis, being from social housing did not significantly moderate the relationship between smoking and having children in the household (Supplementary Table S3).

### Smoking and Quitting-Related Outcomes

Motivation to stop smoking was higher among those with children in the household, and lower among disadvantaged social grades (Table 3). There was no evidence of a moderating effect of social grade on the association between having children in the household and motivation to stop smoking using the Motivation to Stop Scale (Table 3). Sensitivity analyses using a measure of consistent motivation to stop indicated that overall social grade did not moderate consistent motivation to stop among adults with children. Consistent motivation to stop was generally higher in those with than without children, with the exception of social grade D where motivation was similar irrespective of children (Supplementary Table S6 and Figure S2).

There was no evidence of an association between heaviness of smoking and having children in the household, but there was a social gradient with greater HSI among more disadvantaged social grades (Table 3). There was no significant overall moderating effect of social grade on the association between having children in the household and HSI (Table 3).

Weighted prevalence of past-month quit attempts among those with and without children by social grade is displayed in Supplementary Table S2. Past-month quit attempts did not differ significantly according to whether or not there were children in the household, but there was a social gradient with higher prevalence of quit attempts among more disadvantaged social grades compared with AB (Table 3). There was no significant moderating effect of social grade on the association between having children in the household and past-month attempts to quit smoking (Table 3).

The sensitivity analyses using housing tenure as an alternative measure of SEP yielded a pattern of results similar to that in the main analysis (Supplementary Tables S4 and S5).

### The Yearly Trend in Smoking Prevalence Between 2013 and 2019 Among Adults *With* Children in the Household

Between 2013 and 2019, there was a significant negative association between smoking prevalence and year (PR: 0.98, 95% CI 0.97–0.99) in a model adjusted for age, sex, social grade, and region (Supplementary Table S5, Children Model 1). This amounted to a 10.4% (95% CI 15.5–5.1) fall in overall smoking prevalence in 2019 relative to 2013. Age-adjusted trends in smoking prevalence by social grade are presented in Supplementary Figure S3. There was a social gradient in smoking prevalence with those in the most disadvantaged social grade almost five times as likely to smoke compared with the most advantaged social grade AB (Supplementary Table S7, Children Model 1). Smoking declined overall but there was no significant differential time trend according to social grade (Supplementary Table S7, Children Model 2 and Supplementary Figure S1A).

### The Yearly Trend in Smoking Prevalence Between 2013 and 2019 Among Adults *Without* Children in the Household

Between 2013 and 2019, as among those with children, there was a significant negative association between smoking prevalence among adults without children in the household and year (PR: 0.98, 95% CI 0.97–0.99) (Supplementary Table S7, No Children Model 1) which amounted to an 11.4% (95% CI 15.0–7.7) fall in smoking prevalence in 2019 relative to 2013. Age-adjusted trends in smoking prevalence by social grade are presented in Supplementary Figure S3. There was a social gradient in smoking and a differential time trend according to social grade (Supplementary Table S7, No Children Model 2). Smoking prevalence generally declined in all social grades over time, with the exception of social grade D where prevalence appeared stable between 2013 and 2019 (Supplementary Figure S1B).

## Discussion

Between 2013 and 2019 smoking prevalence was higher among adults with and without children from more disadvantaged social grades, despite a higher prevalence of attempts to stop smoking in these groups. Specifically, smoking prevalence was higher among those in social grade E with children in the household compared with those without children. In contrast, across all other social grades having children was associated with lower smoking prevalence. Motivation to stop smoking was higher among those with children in the household, and lower among more disadvantaged social grades. Those from more disadvantaged social grades displayed greater heaviness of smoking, which likely undermined the success of recent quit attempts and motivation to stop. Overall, there was a declining trend in smoking among those with and without children in the household, respectively. There was some evidence for a differential trend among adults without children from social grade D where smoking prevalence appeared stable over time.

Although having children in the household was generally associated with lower smoking prevalence compared with those without children, this was moderated by social grade. In social grade E, smoking prevalence was higher among adults with children compared with those without. This is in stark contrast with all other social grades wherein having children was associated with lower smoking prevalence. Social grade E was estimated to have smoking prevalence almost five times that of the most advantaged group across the time period. In addition to stresses related to both life-course^31^ and current disadvantage (related to lack of opportunity and social support, unstable financial and employment situations,^32^ housing and neighborhood deprivation,^33^ worse health,^34^ and sociocultural smoking norms), the added pressure of providing for a dependent child or children may make smoking more likely and undermine successful quitting.^35^ In contrast, the influence of having children in the household among more advantaged groups may not undermine quitting to the same extent. Moreover, tobacco expenditure has been shown to exacerbate poverty in low-income households in the United Kingdom,^36^ which may itself reinforce the likelihood of persistent smoking among adults and children. In the sensitivity analyses, housing tenure did not moderate the association between smoking and having children in the household. Estimates from the STS show that almost 40% of adults with children in the household live in social housing compared with 29% in other forms of housing.^24^ However, the variable for social housing used includes those renting from the local authority or who belong to a housing association. This measure may display greater socioeconomic diversity than measures of disadvantaged social grade based on occupation and reflect different life stresses that have less influence on smoking behavior when there are children in the household.

The persistent inequalities in smoking reflect lower motivation to stop and greater cigarette addiction among more disadvantaged social grades, despite a greater propensity of attempts to stop smoking in the past month. This suggests that people facing difficult and unequal living circumstances who smoke are just as likely to try and quit compared with more advantaged smokers, but are less likely to quit successfully or avoid relapse.^37^ Lack of success in quitting may then itself undermine motivation to stop and perpetuate “quitting fatigue.” Nonetheless, the higher frequency of recent quit attempts among more disadvantaged social grades presents an opportunity to increase motivation and support those with children to succeed in quitting. Potentially equity-positive approaches to supporting cessation should consider the circumstances that likely determine smoking behavior among priority groups and provide extra support to those who do not quit in response to generally effective individual or population-level interventions. In terms of targeting lower income smoking households, policies, and interventions framed around reducing the exposure of children to secondhand smoke could help to ensure that observed reductions in exposure^6^ are sustained and equity positive. This might include focused feedback on home air quality and smoke-free home policies supported by access to varied and alternative smoking cessation support.^38,39^ Not exclusively, alternatives may include behavioral support and approaches such as financial incentives, targeted interventions, and e-cigarettes.^40–42^

During an active tobacco control policy period between 2013 and 2019, smoking rates generally declined among adults with and without children in England. However, persistent inequalities remain. Without addressing social and material deprivation in the population, health inequalities are inevitable. As demonstrated here, quit attempts are made even among the most disadvantaged groups but because smoking is likely related to a broad range of health and social inequalities, specific smoking cessation interventions and policies (and support for those who do not quit in response), should be nested within broader attempts to reduce socioeconomic inequalities in society across the life-course.^43^

Strengths of the study include the large representative sample of the population in England, allowing a generalizable interrogation of smoking patterns. There are several limitations. First, the use of cross-sectional data limits our ability to infer whether social grade was a cause of smoking among the most disadvantaged group with children in the household. Second, the lack of potentially important unmeasured covariates such as the number and age of children, parental gender, lone parenthood, and other social determinants that may influence the reliability of estimates according to whether or not there were children in the household. It is possible that different levels of these variables explain much of the variance in the association between smoking and having children in the household across the social gradient. Future research should examine how these characteristics influence smoking among those with children, and also what effect changes in smoking behavior have on child smoking uptake and cessation. Third, smoking status was not biochemically verified. Previous research has shown evidence of misreporting of smoking status among pregnant women such that those from less-deprived areas were likely not to report their smoking compared with women from more-deprived areas.^44^ It is possible that similar social desirability biases influenced by SEP exist among reporting of smoking status among those with children. Further work should assess the relationship between stress and smoking and other behaviors such as harmful alcohol consumption and examine the prevalence and correlates of offer and type of support for smoking cessation among disadvantaged households. Finally, there is potential for time-series analyses examining the impact of the COVID-19 pandemic on the social gradient in smoking among adults with children given that 2020 saw a rise in quit attempts (www.smokinginengland.info), and parents spent more time at home with their children.

In conclusion, among the most disadvantaged social grade in England, smoking prevalence was higher in those with children in the household than without. This inequality contrasts with other social grades where smoking prevalence was lower among those with children than without. Motivation to stop smoking was higher among those with children in the household, and lower among more disadvantaged social grades. Those from more disadvantaged social grades were more likely to have higher heaviness of smoking scores, but also more likely to have attempted to quit in the past month. These findings highlight the need to address the persistently high prevalence of smoking among disadvantaged adults with children. Without attention this disparity could exacerbate existing health inequalities due to unequal secondhand smoke exposure, and as the children of smokers go on to smoke themselves at a higher rate than more advantaged individuals in the population. Failing to act on this will undermine progress toward “Smoke free 2030.”

## Supplementary Material

A Contributorship Form detailing each author’s specific involvement with this content, as well as any supplementary data, are available online at https://academic.oup.com/ntr.

ntab211_suppl_Supplementary_Materials

ntab211_suppl_Supplementary_Taxonomy_Form

## Data Availability

The data underlying this article (registration doi:10.17605/OSF.IO/XRDZ7) will be shared on reasonable request to the corresponding author.
