# Supplementary material for: Inequalities in Smoking and Quitting-Related Outcomes Among Adults With and Without Children in the Household 2013–2019: A Population Survey in England
Source: Nicotine Tob Res. 2021 Oct 11;24(5):690–8. doi: 10.1093/ntr/ntab211 (PMC8962729; doi:10.1093/ntr/ntab211)
Supplement: ntab211_suppl_Supplementary_Materials [file ntab211_suppl_supplementary_materials.docx]

**Supplementary materials**

**Table S1: Prevalence of cigarette smoking and past month quit attempts among those with and without children in the household by social grade (weighted data)**

|  | No children in household | Children in household |
| --- | --- | --- |
| **Cigarette smoker** | N = 96,061^1^ | N = 42,572^1^ |
| ***Overall*** | 16,325 (17.0%) | 8,313 (19.5%) |
| ***Social grade*** |  |  |
| AB | 2,450 (9.5%) | 1,034 (8.8%) |
| C1 | 4,126 (15.0%) | 1,691 (15.4%) |
| C2 | 4,094 (20.2%) | 2,205 (23.5%) |
| D | 3,236 (24.0%) | 2,011 (27.9%) |
| E | 2,419 (27.1%) | 1,371 (42.7%) |
| ***Past month quit attempt*** | N = 96,061^1^ | N = 42,572^1^ |
| ***Overall*** | 1,191 (1.2%) | 730 (1.7%) |
| ***Social grade*** |  |  |
| AB | 202 (0.8%) | 95 (0.8%) |
| C1 | 290 (1.1%) | 156 (1.4%) |
| C2 | 269 (1.3%) | 182 (1.9%) |
| D | 238 (1.8%) | 174 (2.4%) |
| E | 193 (2.2%) | 123 (3.8%) |
| ^1^Unweighted n = 138,583 | |  |

**Table S2:** **Association between cigarette smoking prevalence and having children in the household within each social grade***

|  | PR^1^ | 95% CI^2^ | P |
| --- | --- | --- | --- |
| **Social grade** |  |  |  |
| AB | 0.71 | 0.66, 0.77 | **<0.001** |
| C1 | 0.84 | 0.80, 0.89 | **<0.001** |
| C2 | 0.92 | 0.87, 0.96 | **<0.001** |
| D | 0.93 | 0.88, 0.98 | **<0.001** |
| E | 1.11 | 1.05, 1.16 | **<0.001** |

*Referent group is those without children in the household
PR = Prevalence Ratio
CI = Confidence Interval

**Figure S1: Smoking prevalence among adults *with* and *without* children in the household by social grade**


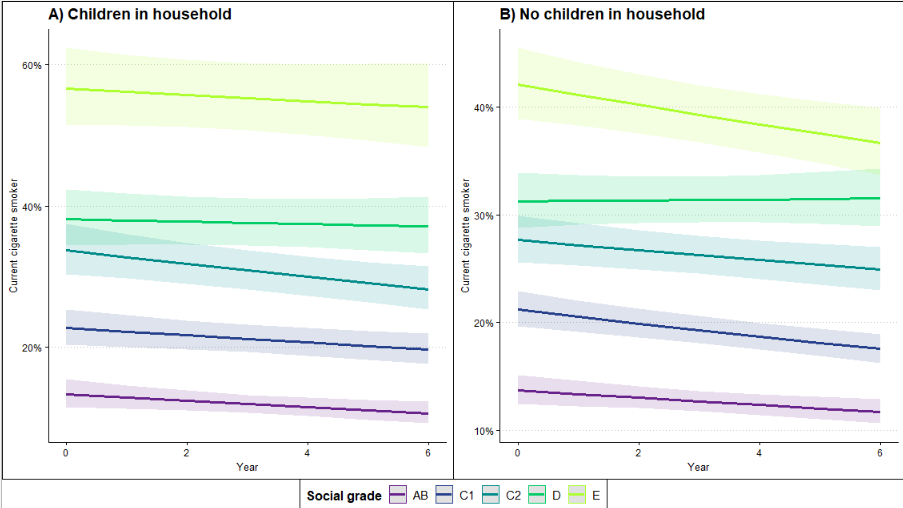


*Models adjusted for age, sex and regio**n*

**Table S3: Association between cigarette smoking prevalence and whether or not there are children in the household and housing tenure**

|  | Model 1 (Without interaction) | | | Model 2 (Interaction) | | |
| --- | --- | --- | --- | --- | --- | --- |
| Variable | OR^1^ | 95% CI^1^ | P | OR^1^ | 95% CI^1^ | P |
| **Children in household** |  |  |  |  |  |  |
| No | — | — |  | — | — |  |
| Yes | 0.82 | 0.79, 0.85 | **<0.001** | 0.82 | 0.79, 0.85 | **<0.001** |
| **Housing tenure** |  |  |  |  |  |  |
| Other | — | — |  | — | — |  |
| Social | 2.95 | 2.85, 3.05 | **<0.001** | 2.94 | 2.82, 3.06 | **<0.001** |
| **Children in household * Housing tenure** |  |  |  |  |  |  |
| Yes * Social |  |  |  | 1.01 | 0.94, 1.08 | 0.85 |
| ^1^OR = Odds Ratio, CI = Confidence Interval  *Models adjusted for age, sex, region and year* | | | | | | |
|  | | | | | | |

**Table S4: Associations between smoking and quitting behaviour and whether or not there were children in the household and housing tenure (without interaction models)**

|  | Motivation to stop smoking | | | Heaviness of smoking | | | Past month quit attempt | | |
| --- | --- | --- | --- | --- | --- | --- | --- | --- | --- |
| Variable | PR^1^ | 95% CI^2^ | P | PR^1^ | 95% CI^2^ | P | PR^1^ | 95% CI^2^ | P |
| **Children in household** |  |  |  |  |  |  |  |  |  |
| No | — | — |  | — | — |  | — | — |  |
| Yes | 1.16 | 1.09, 1.25 | **<0.001** | 0.95 | 0.89, 1.03 | 0.23 | 0.98 | 0.88, 1.08 | 0.64 |
| **Housing tenure** |  |  |  |  |  |  |  |  |  |
| Other | — | — |  | — | — |  | — | — |  |
| Social | 0.93 | 0.87, 0.99 | **0.032** | 1.71 | 1.61, 1.82 | **<0.001** | 2.41 | 2.19, 2.65 | **<0.001** |
| ^1^PR = Prevalence Ratio | | | | | | | | | |
| ^2^CI = Confidence Interval | | | | | | | | | |
| *Models adjusted for age, sex, region and year* | | | | | | | | | |

**Table S5: Associations between smoking and quitting behaviour and whether or not there were children in the household and housing tenure (interaction models)**

|  | Motivation to stop smoking | | | Heaviness of smoking | | | Past month quit attempt | | |
| --- | --- | --- | --- | --- | --- | --- | --- | --- | --- |
| Variable | PR^1^ | 95% CI^2^ | P | PR^1^ | 95% CI^2^ | P | PR^1^ | 95% CI^2^ | P |
| **Children in household** |  |  |  |  |  |  |  |  |  |
| No | — | — |  | — | — |  | — | — |  |
| Yes | 1.17 | 1.08, 1.26 | **<0.001** | 0.96 | 0.87, 1.06 | 0.39 | 0.96 | 0.85, 1.09 | 0.55 |
| **Housing tenure** |  |  |  |  |  |  |  |  |  |
| Other | — | — |  | — | — |  | — | — |  |
| Social | 0.93 | 0.85, 1.02 | 0.11 | 1.71 | 1.59, 1.84 | **<0.001** | 2.37 | 2.09, 2.68 | **<0.001** |
| **Children in household * Housing tenure** |  |  |  |  |  |  |  |  |  |
| Yes * Social | 1.00 | 0.88, 1.14 | 0.98 | 0.99 | 0.87, 1.13 | 0.92 | 1.04 | 0.86, 1.26 | 0.69 |
| ^1^PR = Prevalence Ratio | | | | | | | | | |
| ^2^CI = Confidence Interval | | | | | | | | | |
| *Models adjusted for age, sex, region and year* | | | | | | | | | |

**Table S6: Associations between consistent motivation to stop smoking and whether or not there were children in the household and social grade**

|  | Model 1 (Without interaction) | | | Model 2 (Interaction) | | |
| --- | --- | --- | --- | --- | --- | --- |
| Variable | PR^1^ | 95% CI^2^ | P | PR^1^ | 95% CI^2^ | P |
| **Children in household** |  |  |  |  |  |  |
| No | — | — |  | — | — |  |
| Yes | 1.13 | 1.10, 1.17 | **<0.001** | 1.15 | 1.07, 1.23 | **<0.001** |
| **Social grade** |  |  |  |  |  |  |
| AB | — | — |  | — | — |  |
| C1 | 0.94 | 0.90, 0.98 | **0.003** | 0.94 | 0.89, 0.99 | **0.013** |
| C2 | 0.86 | 0.82, 0.89 | **<0.001** | 0.86 | 0.81, 0.90 | **<0.001** |
| D | 0.84 | 0.80, 0.88 | **<0.001** | 0.88 | 0.83, 0.93 | **<0.001** |
| E | 0.89 | 0.85, 0.93 | **<0.001** | 0.88 | 0.83, 0.93 | **<0.001** |
| **Children in household * Social grade** |  |  |  |  |  |  |
| Yes * C1 |  |  |  | 1.01 | 0.92, 1.10 | 0.87 |
| Yes * C2 |  |  |  | 1.00 | 0.91, 1.09 | 0.97 |
| Yes * D |  |  |  | 0.89 | 0.81, 0.98 | **0.022** |
| Yes * E |  |  |  | 1.03 | 0.93, 1.13 | 0.57 |
| ^1^PR = Prevalence Ratio  ^2^CI = Confidence Interval | | | | | | |
| *Models adjusted for age, sex, region and year* | | | | | | |

**Figure S2: Predicted probabilities of consistent motivation to stop smoking among adults with children in the household by Social grade**


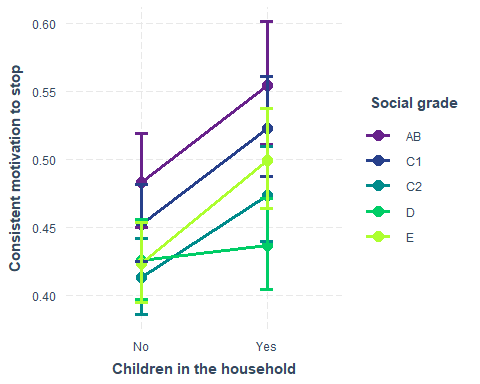


*Model adjusted for age, sex, region and year*

**Table S7: Association between smoking prevalence and social grade and year among adults with children in the house**

|  | Children Model 1 (Without interaction) | | | Children Model 2 (Interaction) | | | No children Model 1 (Without interaction) | | | No children Model 2 (Interaction) | | |
| --- | --- | --- | --- | --- | --- | --- | --- | --- | --- | --- | --- | --- |
|  | (χ2(4)=1951.7, P<0.001) | | | (χ2(4)=5.6, P=0.23) | | | (χ2(4)=2527.8, P<0.001) | | | (χ2(4)=10.6, P=0.03) | | |
| Variable | PR^1^ | 95% CI^2^ | P | PR^1^ | 95% CI^2^ | P | PR^1^ | 95% CI^2^ | P | PR^1^ | 95% CI^2^ | P |
| **Year of survey** | 0.98 | 0.97, 0.99 | **<0.001** | 0.96 | 0.93, 1.00 | **0.029** | 0.98 | 0.97, 0.99 | **<0.001** | 0.97 | 0.95, 0.99 | **0.012** |
| **Social grade** |  |  |  |  |  |  |  |  |  |  |  |  |
| AB | — | — |  | — | — |  | — | — |  | — | — |  |
| C1 | 1.78 | 1.64, 1.92 | **<0.001** | 1.70 | 1.48, 1.97 | **<0.001** | 1.52 | 1.45, 1.60 | **<0.001** | 1.54 | 1.41, 1.69 | **<0.001** |
| C2 | 2.59 | 2.40, 2.80 | **<0.001** | 2.52 | 2.20, 2.91 | **<0.001** | 2.07 | 1.97, 2.18 | **<0.001** | 2.02 | 1.84, 2.21 | **<0.001** |
| D | 3.15 | 2.91, 3.41 | **<0.001** | 2.86 | 2.48, 3.29 | **<0.001** | 2.47 | 2.34, 2.60 | **<0.001** | 2.28 | 2.07, 2.50 | **<0.001** |
| E | 4.62 | 4.27, 5.00 | **<0.001** | 4.24 | 3.70, 4.88 | **<0.001** | 3.10 | 2.95, 3.27 | **<0.001** | 3.07 | 2.80, 3.36 | **<0.001** |
| **Year of survey * Social grade** |  |  |  |  |  |  |  |  |  |  |  |  |
| Year of survey * C1 |  |  |  | 1.01 | 0.97, 1.05 | 0.51 |  |  |  | 1.00 | 0.97, 1.02 | 0.69 |
| Year of survey * C2 |  |  |  | 1.01 | 0.97, 1.05 | 0.70 |  |  |  | 1.01 | 0.98, 1.03 | 0.49 |
| Year of survey * D |  |  |  | 1.03 | 0.99, 1.07 | 0.10 |  |  |  | 1.03 | 1.00, 1.06 | **0.036** |
| Year of survey * E |  |  |  | 1.03 | 0.99, 1.07 | 0.14 |  |  |  | 1.00 | 0.98, 1.03 | 0.78 |
| ^1^PR = Prevalence Ratio |  |  |  |  |  |  |  |  |  |  |  |  |
| ^2^CI = Confidence Interval |  |  |  |  |  |  |  |  |  |  |  |  |
| *Models adjusted for age, sex and region* | | | | | | | | | | | | |

**Figure S3: Age-adjusted trends in smoking prevalence among adults *without* and *with* children in the household 2013-2019**

**
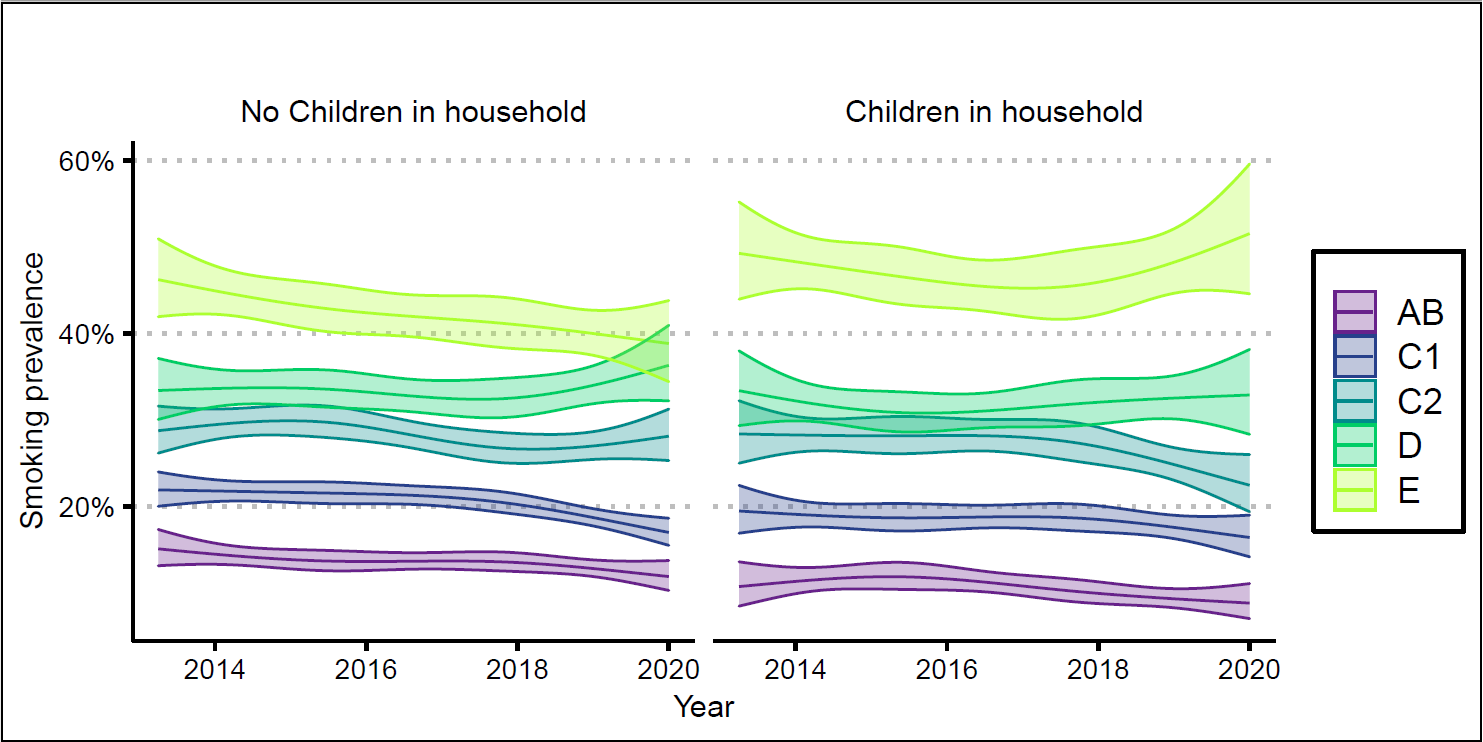
**

*Fitted log-binomial regression of smoking prevalence as a function of year and children in the household, while holding age constant. Shaded bands represent 95%CIs. To allow for non-linear trends, year was modelled using a natural cubic spline with five knots, each placed at equally-spaced quantiles of the data (Harell 2015).*

**Power analysis to guide decision on interaction models**

Prior to running the models, we conducted a power analysis using simulations based on data from previous years of the smoking toolkit study (2006-2012). This allowed us to check whether we would have the statistical power to reliably estimate a possible three-way interaction having children*SEP*year (continuous). For each model, we had 80% power to detect effects of the size highlighted (using p<.05).

For each model, we have 80% power to detect effects of the size highlighted below (using p<.05).

Sample: All adults

**Model** **a**: Year*Children in household (Y/N)*social grade (five groups AB, C1, C2, D, E) triple interaction

**Outcome**: Smoking (yes/no)

**Predictors**: Year (continuous); Children in household; Social grade; Year*Child in household interaction; Social grade*Year interactions; Social grade*Year*Children in household triple interactions.

**Powered for relative differences of:** RR > 1.07

**Summary**: 80% power to detect >7% relative differences, depending on social grade, in how much greater/lower the yearly rate of change in smoking prevalence is between those with vs without children.

**Model b**: Year*Children in household (Y/N)*Social grade (dichotomised ABC1, C2DE) triple interaction

**Outcome:**  Smoking (yes/no)

**Predictors:** Year (continuous); Children in household; Social grade (ABC1); Year*Children in household interaction; Social grade ABC1*Year interaction; Social grade ABC1*Year*Children in household triple interaction.

**Powered for relative differences of:** RR > 1.038

**Summary**: 80% power to detect >3.8% differences, depending on social grade, in how much greater/lower the yearly rate of change in smoking prevalence is between those with vs without children.

NB: The absolute percentage point differences are and will vary based on actual prevalence we find in each group.

**Figure S4: Approximating the minimum effect size of the triple interaction that we have 80% power to detect.**


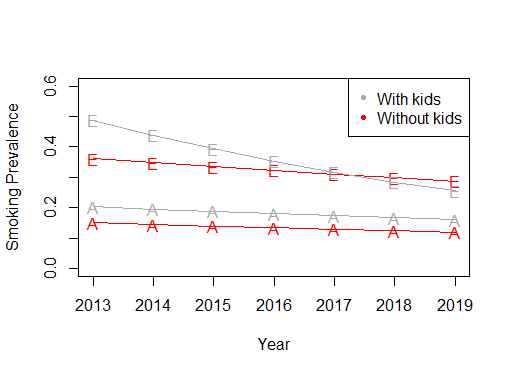


This figure shows the minimum effect size for the triple interaction between social grade (ABC1/C2DE), children in home (Y/N), and year (continuous) that we are able to detect with 80% power. “A” in the graph represents ABC1 and “E” represents C2DE. This is computed using simulations with STS 2006-2012 data.
